# Transparency in Reporting Observational Studies: Reflections after a Year

**DOI:** 10.1371/journal.pmed.1001896

**Published:** 2015-10-27

**Authors:** The PLOS Medicine Editors

## Abstract

The *PLOS Medicine* Editors take stock of changes in the reporting of observational studies following our new transparency guidelines from August 2014.

As an open-access journal committed to ameliorating the major challenges to human health worldwide, *PLOS Medicine* strives to publish transparent papers that researchers can replicate and that clinicians and policymakers can accurately interpret. As editors, our best levers may be journal policies intended to strengthen the reporting of the studies that we publish. Just over a year ago, we stepped up our reporting requirements—and our requests to authors—in order to shine a brighter light on the methods used in observational studies published in *PLOS Medicine*. Seventeen published observational studies later, as we extend our thanks to authors and reviewers for their support and patience during implementation of the new requirements, we think this is a good time to take stock of the early effects.

Our August 2014 editorial [1] kicked off the four new requirements, modeled on the framework for clinical trials. These requirements are as follows:

- Checklist-documented adherence to the Strengthening the Reporting of Observational Studies in Epidemiology (STROBE) guidelines for cohort, case-control, or cross-sectional studies [2] and to the Standards for Reporting of Diagnostic Accuracy (STARD) guidelines for studies of diagnostic accuracy [3]
- Commitment to data sharing via provision of a Data Availability Statement describing how researchers can obtain the study’s underlying data. This policy is shared across all *PLOS* journals [4,5]
- Specification in the paper of planned versus completed analyses
- Provision of any prospective analysis plan used in designing the study

Our goal, as ever, is to provide the medical community with the information needed to inform patient care, health policy, and future studies. Our additional hopes are that researchers themselves will benefit from a wider culture of improving the transparency of observational studies and that transparency will justify and normalize the natural changes to protocol that research, and peer review, entail.

Have *PLOS Medicine’s* observational papers changed for the better? This is ultimately for *PLOS Medicine* readers to determine, and we would be happy to hear your feedback. In the meantime, a look back through the published observational studies that were submitted after the policy took effect [6–22] provides some insight (albeit without the benefit of STROBE or a prospective analysis plan in this case). All “data” are from *PLOS Medicine* papers, which are freely available on our website.

## The Elusive Prospective Analysis Plan

We knew that our call for prospective analysis plans for observational studies would be the most aspirational of the four requests; creating a date-stamped plan is de rigueur for clinical trials but not for observational studies. Indeed, such plans have been few among our published observational studies this past year. One strong exception publishes this month; Ayse Ercumen and colleagues’ matched cohort study, examining the benefits of a piped water supply in urban India, provides a full date-stamped protocol (dated upon submission to the Institutional Review Board [IRB]), along with a document listing all changes post hoc and the reasons for them [19]. Overall, few *PLOS Medicine* authors prepare a date-stamped protocol for observational studies, even if the study is hypothesis driven and analyses are planned in advance of data collection. However, our authors are resourceful: applications for IRB approval, funding, or data use are sometimes included as supporting information when no official protocol exists. While we hope authors will continue to provide these forms of documentation, we also note that analyses and variables are often tentative at the funding or data application stage. We therefore encourage researchers to consider generating records of planned analyses, to document which analyses were driven by hypotheses and which, appropriately, by new and interesting data.

## Telling the Whole Story

Where no prespecified analysis plan exists, we ask that authors indicate in the paper which analyses are prespecified and which are exploratory. When analyses change from the original plan, we ask authors to indicate why. Reviewer fingerprints, along with other evidence of considered changes, have begun to appear in published *PLOS Medicine* observational studies. As examples, in three cohort studies, peer review resulted in a change in the index used to measure poverty or wealth [14], an additional source of potential confounding was integrated based on peer review [9], and study authors added a new hypothesis to the study design once participant enrollment exceeded expectation [16]. Study authors may benefit from acknowledging guidance from peer review, as readers may reasonably infer that reviewers’ proposed post hoc analyses are pointed and rational, rather than evidence of “data dredging” or “cherry picking.” In the most recent observational study published in the journal, Aurélie Jeandron and colleagues’ time-series regression showing an association between water supply interruptions and incidence of suspected cholera in the Democratic Republic of the Congo, the authors provide, as supporting information, a full timeline of the analysis, including correction of a model and revision of a sensitivity analysis in response to reviewer comments at *PLOS Medicine* [20]. An ancillary benefit of “telling the whole story” is that the successes of peer review, a process currently under scrutiny, are brought into the open.

## Sharing the Data

Of the four transparency measures, the commitment to data availability inspires perhaps the most—and most interesting—conversations among editors, authors, and reviewers. The Data Availability Policy is a *PLOS*-wide effort that was implemented six months before we introduced these new requirements for observational studies at *PLOS Medicine* [4]. Data Availability Statements direct researchers wishing to replicate findings or further analyze data to several possible sources, including the article and supporting information, a third-party source (either an email address or a website; the data contact person cannot be a study author), a data repository, or some combination of the three. In our *PLOS Medicine* cohort of 17 post-transparency observational studies, the underlying data for seven papers are reported as accessed through a third-party data source. Several of these use data from large cohort studies, such as the United Kingdom Clinical Practice Research Datalink, The Swedish National Study on Aging and Care in Kungsholmen, and the United States Southern Community Cohort Study. We ask that authors who use such datasets also indicate any requirements, contracts, or limitations associated with data use, in order that researchers can quickly assess the resources they will need to obtain the data. Increasingly (here, in six papers), Data Availability Statements indicate that observational data are contained within the paper and supporting information files. A few authors (those of four of these papers) reference deposited data—in Dryad in one case [9], Open Science Framework for another [19], and, for two other recent papers [8,21], in the London School of Hygiene and Tropical Medicine (LSHTM) Data Compass and the University of California Curation Center (UC3), both of which are digital repositories for data generated by these institutions and their collaborators.

## Going Forward

As transparent practices gain ground among academic publishers, we observe that our academic editors and reviewers, like us, seek reporting checklists, prospective plans, and study data at early stages in the manuscript evaluation process. Some use the reporting checklist as a guide to the paper; others analyze the data themselves to verify conclusions. Many are concerned that exploratory analyses might be pitched as hypothesis driven. To speed the evaluation of observational research submissions to the journal, we encourage authors to provide the needed materials upon submission of the manuscript. Doing so will help all editors and reviewers who assess the manuscript to work with the authors towards articles that meet a key criterion of suitability for publication in *PLOS Medicine*—transparent reporting sufficient for replication and interpretation.

As always, we’d love to hear more from readers and researchers about our ongoing efforts to illuminate data and analysis in observational studies.

## Abbreviations

IRB: Institutional Review Board
LSHTM: London School of Hygiene and Tropical Medicine
STARD: Standards for Reporting of Diagnostic Accuracy
STROBE: Strengthening the Reporting of Observational Studies in Epidemiology
UC3: University of California Curation Center
